# Switchable LED-based laparoscopic multispectral system for rapid high-resolution perfusion imaging

**DOI:** 10.1117/1.JBO.28.12.126002

**Published:** 2023-12-13

**Authors:** Annekatrin Pfahl, Süleyman T. Polat, Hannes Köhler, Ines Gockel, Andreas Melzer, Claire Chalopin

**Affiliations:** a Leipzig University, Faculty of Medicine, Innovation Center Computer Assisted Surgery, Leipzig, Germany; b University Hospital of Leipzig, Department of Visceral, Transplant, Thoracic, and Vascular Surgery, Leipzig, Germany; c University of Dundee, School of Medicine, Institute for Medical Science and Technology, Dundee, United Kingdom; d University of Applied Sciences and Arts, Faculty of Engineering and Health, Göttingen, Germany

**Keywords:** multispectral imaging, tissue hemoglobin content, tissue oxygenation, perfusion, laparoscope, occlusion study

## Abstract

**Significance:**

Multispectral imaging (MSI) is an approach for real-time, quantitative, and non-invasive tissue perfusion measurements. Current laparoscopic systems based on mosaic sensors or filter wheels lack high spatial resolution or acceptable frame rates.

**Aim:**

To develop a laparoscopic system for MSI-based color video and tissue perfusion imaging during gastrointestinal surgery without compromising spatial or temporal resolution.

**Approach:**

The system was built with 14 switchable light-emitting diodes in the visible and near-infrared spectral range, a 4K image sensor, and a 10 mm laparoscope. Illumination patterns were created for tissue oxygenation and hemoglobin content monitoring. The system was calibrated to a clinically approved laparoscopic hyperspectral system using linear regression models and evaluated in an occlusion study with 36 volunteers.

**Results:**

The root mean squared errors between the MSI and reference system were 0.073 for hemoglobin content, 0.039 for oxygenation in deeper tissue layers, and 0.093 for superficial oxygenation. The spatial resolution at a working distance of 45 mm was 156  μm. The effective frame rate was 20 fps.

**Conclusions:**

High-resolution perfusion monitoring was successfully achieved. Hardware optimizations will increase the frame rate. Parameter optimizations through alternative illumination patterns, regression, or assumed tissue models are planned. Intraoperative measurements must confirm the suitability during surgery.

## 1 Introduction

In visceral surgery, tissue oxygenation is a critical factor for anastomotic healing.^1^ To define an optimal anastomotic position in well-oxygenated areas, the supportive value of hyperspectral imaging (HSI) was examined in several studies that were recently reviewed.^2^ Although objective, non-invasive, and quantifiable, especially in contrast to the often-used fluorescence angiography with indocyanine green, HSI lacks high temporal resolution. Fast image registration from static hyperspectral images to standard color videos^3^ can compensate for this disadvantage to some extent. In minimally invasive procedures with rigid or flexible endoscopes, multispectral imaging (MSI), due to its video-rate capability, is particularly important when observing areas with movement such as peristalsis or near the heart, for continuous monitoring of perfusion changes or when moving the endoscope.

The potential of MSI to support surgeons during the assessment of tissue oxygenation has already been shown. A laparoscopic MSI system with a xenon light source and a filter wheel to acquire eight narrow spectral bands provided a color image, hemoglobin, and oxygenation maps in concurrence with the clinical assessment for seven patients undergoing colorectal surgery. The system covered the spectral range from 470 to 645 nm and had a frame rate of three frames per second (fps).^4^ Another filter wheel-based system with eight filters for open surgery was used in combination with a deep learning architecture and Monte Carlo simulations to improve the accuracy of provided perfusion parameters in comparison to oxygenation estimations based on the modified Beer–Lambert law. The system covered the visible spectral range (470 to 700 nm) and captured raw data at a frame rate of 2 fps.^5^

To further increase the temporal resolution, snapshot systems using mosaic sensors are of interest. Unlike spectral or spatial scanning systems (see Refs. 6 and 7 for an extended summary of HSI and MSI acquisition techniques), reflectance spectra over an entire surface are acquired at a glance. In Ref. 8, the xiSpec camera (XIMEA GmbH, Münster, Germany) was used to acquire raw spectral images at 16 wavelengths in the range from 450 to 650 nm at 50 fps. Due to image postprocessing and temporal averaging of consecutive raw images, the effective frame rate of calculated oxygenation maps was 1.4 fps. The main drawback of snapshot systems is their spatial resolution resulting from the reduced number of effective pixels for each spectral band (512×272 effective pixels compared to 2048×1088 sensor pixels^8^).

Another alternative to mosaic sensors or filter wheel approaches is a tunable light source, e.g., switchable light-emitting diodes (LEDs), as presented as non-laparoscopic systems in Refs. 9 and 10. In combination with 4K or full HD monochromatic or color sensors, higher spatial resolutions are achievable than with mosaic sensors. Furthermore, effective frame rates ≥24  fps for real-time visualizations are possible through the fast switching of LEDs. In contrast to filter wheels, a subclass of available spectral bands can be used, enabling distinct application-related illuminations without any hardware adaptations. Therefore, the development of a laparoscopic MSI system based on switchable LEDs for perfusion monitoring has been the aim of this work. Multispectral illumination patterns and data-processing pipelines for simultaneous high-resolution color and tissue oxygenation or hemoglobin content videos have been implemented and initially evaluated in a laboratory study with healthy volunteers.

## 2 Material and Methods

This section includes descriptions of the developed MSI system, technical evaluations, a reference HSI system, and performed methods for measuring tissue oxygenation and hemoglobin content.

### 2.1 Setup of the Laparoscopic MSI System

**Fig. 1:**
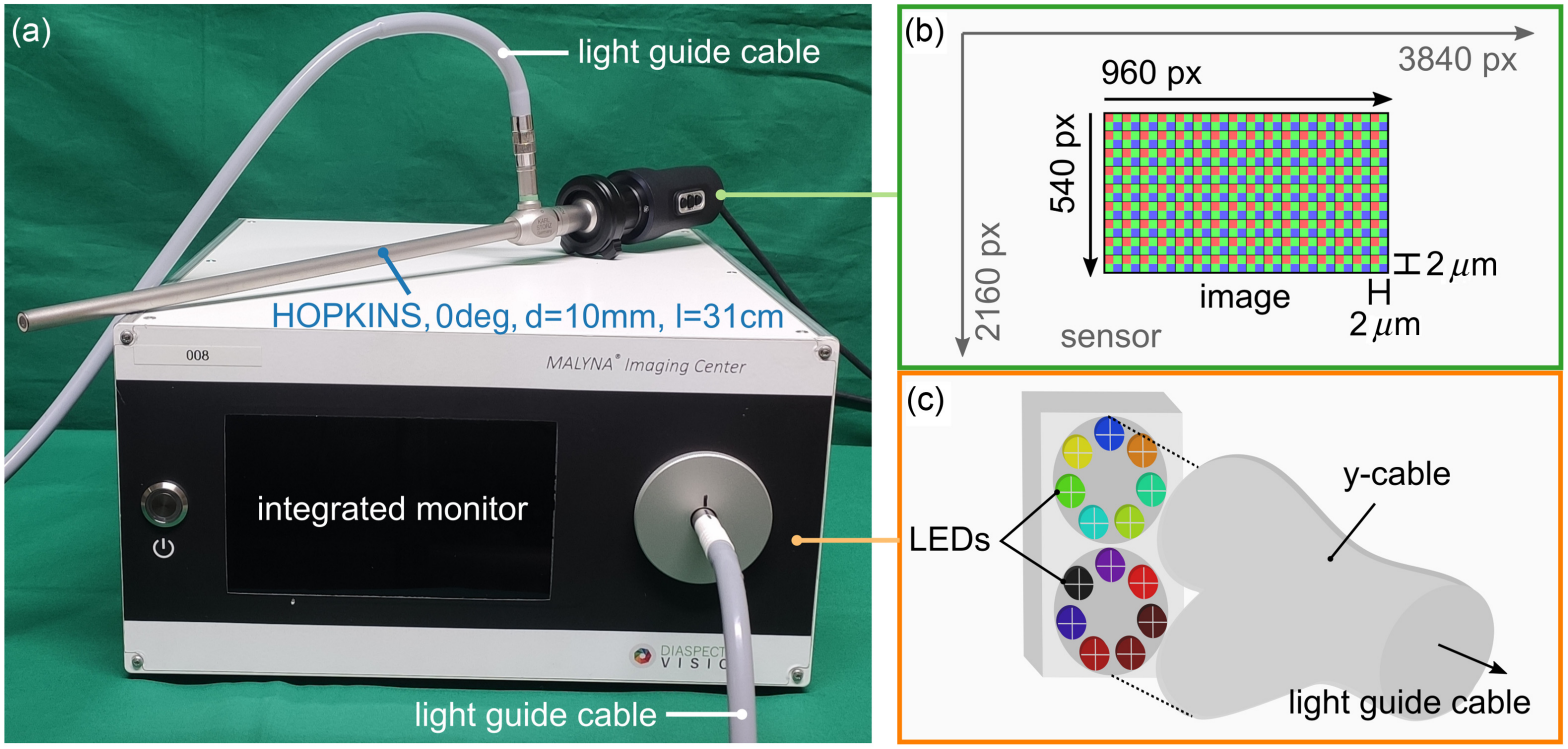
(a) Setup of the MSI laparoscope with the imaging center, the hand-held camera, a HOPKINS optics (KARL STORZ SE & Co. KG, Tuttlingen, Germany), and a light guide cable (Faseroptik Jena, Jena, Germany). (b) A color CMOS sensor inside the camera housing with high sensitivity in the NIR range (OMNIVISION, Santa Clara, United States) serves for imaging. Although it is 4K capable, raw images currently have a size of $960\times 540\;{\mathrm{px}}^{2}$. A single pixel covers an area of $2\times 2\;\mu m^{2}$. (c) The light source (Diaspective Vision GmbH, Am Salzhaff-Pepelow, Germany) contains 14 individually switchable LEDs in 2 blocks of 7 LEDs each. The light couples into the light guide cable via a y-piece.

The overall system [Fig. 1(a)] consists of a hand-held camera unit and a light source and processing unit called the imaging center (Diaspective Vision GmbH, Am Salzhaff-Pepelow, Germany). The latter includes 14 narrow-band LEDs covering the spectral range from 405 to 960 nm (nominal wavelengths). LEDs can be triggered individually to create distinct illumination patterns. The light couples into a light guide cable (diameter = 4.8 mm, length = 230 cm, transmittance = 37% to 57% from 400 to 1000 nm, Faseroptik Jena, Jena, Germany) via a y-piece, which is directly located in front of the LED boards [Fig. 1(c)] and guides the light to a rigid laparoscope (HOPKINS broadband, 0 deg, diameter = 10 mm, length = 31 cm, KARL STORZ SE & Co. KG, Tuttlingen, Germany). The laparoscope is connected to the camera housing via an objective lens to a C-mount adapter. Inside the camera [Fig. 1(b)], a 4K color CMOS sensor (OMNIVISION, Santa Clara, United States) detects light in the visible (VIS) and near-infrared (NIR) range at up to 60 fps. After transfer to the internal processing unit via ethernet, raw data are currently available with 40 fps and an image size of $960\times 540\;{\mathrm{px}}^{2}$. The image depth is 10 bits. With an objective lens with a focal length of 25 mm and specific illumination patterns (Sec. 2.4.1), the spatial resolution of the system is 156  μm at a working distance of 45 mm with a Michelson contrast >20% (Fig. S1 in the Supplementary Material). The field of view at a working distance of 45 mm is $∼4.2\times 2.35\;{\mathrm{cm}}^{2}$.

### 2.2 Data Acquisition and Technical Evaluations

The image acquisition relies on spectral scanning in reflectance mode. There is a synchronization between the light source and the image sensor. LEDs emit continuously, flash simultaneously, or alternately depending on the selected mode. A “sequence” describes which LEDs light up and for how long during the exposure of up to three red-green-blue (RGB) “images” captured in a loop by the image sensor. An image is assigned with the “image index” 1, 2, or 3. A “frame” is any image of a sequence regardless of the index and indicates the total number of recorded images.

Spectral separation of signals from different LEDs is possible by alternating lighting or reading out the individual color channels of the image sensor. The latter is only feasible if simultaneously used LEDs do not contribute to the same color channel. The emission spectra of some LEDs overlap with more than one filter curve of the image sensor. This effect leads to more than 14 available spectral bands, i.e., effective wavelengths. The effective spectrum of the presented system contains 16 relevant effective wavelengths resulting from the multiplication of the emission spectra of the 14 LEDs with each filter curve of the image sensor (RGB filter) as well as the transmission curves of the light guide and optics. The effective peak wavelengths and full widths at half maximum (FWHM) are listed in Table 1.

**Table 1:** Effective peak wavelengths of the MSI system and corresponding FWHM.

Peak wavelength in nm	404	426	450	503	519	532	537	580	590	600	635	663	762	811	863	957
FWHM in nm	12	16	21	32	38	75	75	57	84	49	17	20	32	36	49	43

#### 2.2.1 Light source behavior

A significant part of the overall system is the temporal behavior of the light source. If the synchronization between the light source and image sensor does not work properly, the lighting shows a temporal delay, or intensities highly fluctuate over time, the reliable acquisition of spectral data is not possible. At first, the switching behavior of all LEDs was examined. The considered LED remained off within the first and third images and was on within the second one over 30 frames (10 frames per image index). The mean pixel value was calculated for each frame and averaged over all frames with the same image index [Fig. S2(a) in the Supplementary Material]. Ideally, this is zero for the first and third images and maximal for the second image. Afterward, the considered LED was continuously on for one hour. The mean pixel value was calculated for one frame per minute. Ideally, this is constant over time. The image sensor level control was set to 80%±2% in all cases by adjusting the time of illumination and pulse width modulation of each LED, and the gain of the sensor at a working distance of 45 mm.

#### 2.2.2 Signal-to-noise ratio

The signal-to-noise ratio (SNR) was determined for each effective wavelength according to Eq. (1) and spatially resolved at a working distance of 45 mm. The image sensor level control was set to 80%±2%. A comparison to a reference HSI system (Sec. 2.3) served to assess the suitability of the system to image both parameters. SNRs were also related to final tissue oxygenation and hemoglobin content calculations

$$\mathrm{SNR}=\frac{1}{N}\overset{N}{\underset{i=1}{\sum }}20\;\log\;\frac{\overset{¯}{v}}{\left|v_{i}-\overset{¯}{v}\right|},$$ (1)

with N=1000: number of acquired frames, $v_{i}$: mean pixel value of frame i, and $\overset{¯}{v}$: mean pixel value averaged over 1000 frames.

### 2.3 Reference HSI System

The TIVITA Mini (Diaspective Vision GmbH, Am Salzhaff-Pepelow, Germany) is a clinically approved push-broom HSI system^11^ used for technical and application-related comparisons to the presented MSI system. It provides information about superficial tissue oxygenation (StO2), oxygenation in deeper tissue layers of up to about 5 mm (NIR PI short for near-infrared perfusion index), and tissue hemoglobin content (THI short for tissue hemoglobin index).^12^ It covers a similar spectral range from 500 to 1000 nm and is also a laparoscopic system. Results of the first *ex vivo* and *in vivo* studies during gastrointestinal surgery are available in Refs. 13 and 14, respectively.

Physiological tissue parameters are calculated from a hypercube [Eq. (2) based on Ref. 12], which includes reflectances calculated according to Eq. (3) in the above-mentioned spectral range for an image size of $640\times 480\;{\mathrm{px}}^{2}$. Data acquisition and parameter calculation take about 7 s. The spatial resolution of the system is 320  μm at a working distance of 50 mm using an objective lens with a focal length of 14 mm and green light (500 nm).^11^ Here, an objective lens with a focal length of 22 mm was used

$$P_{\mathrm{HSI}}=\frac{f(R(x,y,s_{1}))}{f(R(x,y,s_{2}))},$$ (2)

with $P_{\mathrm{HSI}}$: HSI parameter value; f(R): reflectance value after postprocessing using the TIVITA proprietary software; x,y: spatial coordinates; and $s_{1},s_{2}$: spectral ranges

$$R=\frac{I_{\text{raw}}-I_{D}}{I_{w}-I_{D}},$$ (3)

with R: reflectance, $I_{\text{raw}}$: raw intensity image, $I_{D}$: dark reference image (lights off), and $I_{W}$: white reference image.^6^

### 2.4 Tissue Hemoglobin Content and Oxygenation Monitoring

Three multispectral illumination patterns were developed for tissue hemoglobin content and oxygenation measurements. These sequences contain two images each to calculate one physiological parameter and an additional standard color (RGB) image. The superficial tissue oxygenation called StO2 in the reference HSI system is referred to as ${\mathrm{i.StO}2}_{\sup}$. Oxygenation in deeper tissue layers is called i.StO2 and is compared to the NIR PI of the reference system. Tissue hemoglobin content, i.tHb, should correspond to the THI.

#### 2.4.1 Wavelength selection

**Fig. 2:**
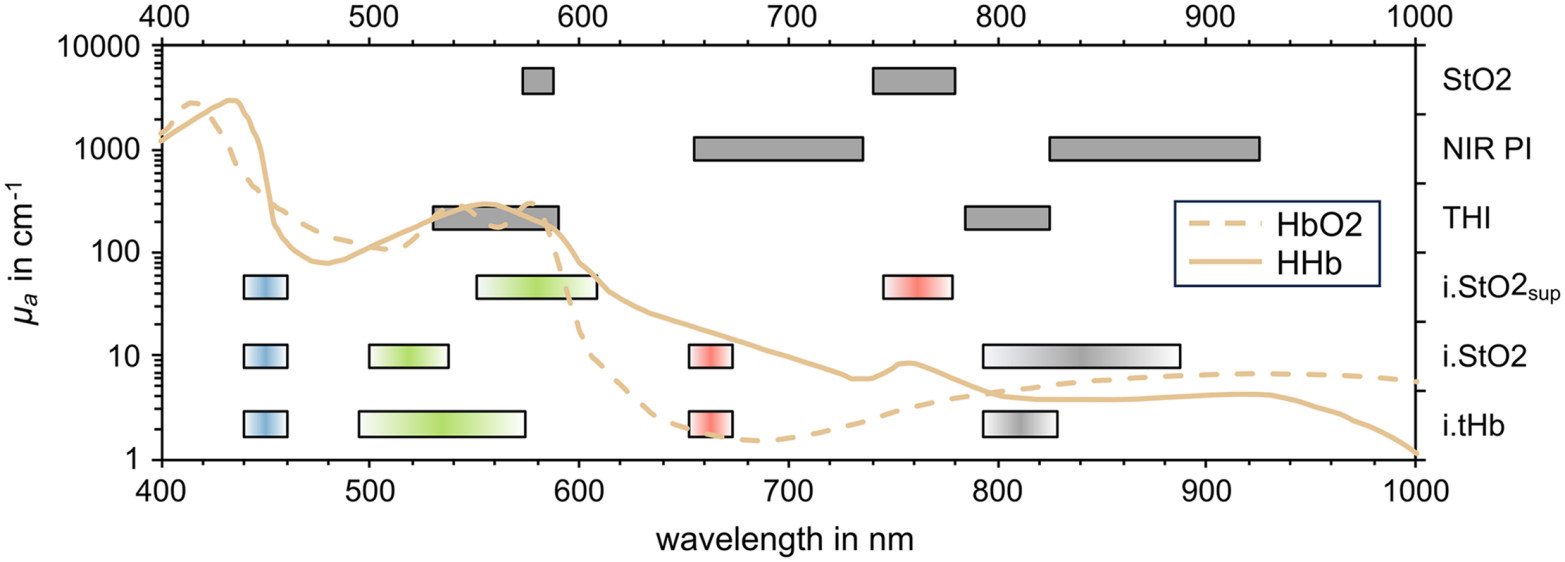
Selected spectral bands for the multispectral illumination to visualize a color image and to determine i.tHb, i.StO2, and ${\mathrm{i.StO}2}_{\sup}$ compared to the absorption spectra of oxygenated (HbO2) and deoxygenated (HHb) hemoglobin. The color gradient symbolizes the wavelength-dependent light intensity of the LEDs used. Spectral ranges used in the reference hyperspectral system to calculate corresponding THI, NIR PI, and StO2 are also shown.

The wavelength selection from 16 available effective wavelengths for the MSI sequences relied on the absorption characteristics of oxygenated (${\mathrm{HbO}}_{2}$) and deoxygenated (HHb) hemoglobin.^15^ Spectral ranges with high absorption coefficients $\mu_{a}$ were of interest for i.tHb. Therefore, reflectances from green light were related to spectral information close to the isosbestic point at 800 nm where ${\mathrm{HbO}}_{2}$ and HHb have small $\mu_{a}$. Blue and red LEDs were added to the sequence for an additional RGB image (Fig. 2).

Two spectral ranges were selected where ${\mathrm{HbO}}_{2}$ and HHb show opposing absorption characteristics for both tissue oxygenation sequences. Like in standard pulse oximetry, the calculated reflectance ratio (RR) [Eq. (4)] between them becomes large if much ${\mathrm{HbO}}_{2}$ is present, i.e., the oxygenation is high, or low if much HHb is present, i.e., the oxygenation is low. Green light that has a small penetration depth and NIR light were used for the superficial tissue oxygenation ${\mathrm{i.StO}2}_{\sup}$. Lighting is performed in the red and NIR spectral range to measure oxygenation in deeper tissue layers (i.StO2). Further LEDs were added for additional RGB images (Fig. 2).

A comparison to the spectral ranges used in the reference system to calculate the THI, NIR PI, and StO2 (also shown in Fig. 2) was conducted to initially prove the developed sequences

$${\mathrm{RR}}_{\mathrm{MSI}}=\frac{f^{\prime }(R(x,y,s_{1}^{\prime }))}{f^{\prime }(R(x,y,s_{2}^{\prime }))},$$ (4)

with ${\mathrm{RR}}_{\mathrm{MSI}}$: MSI reflectance RR, $s_{1}^{\prime },s_{2}^{\prime }$: spectral ranges.

#### 2.4.2 Occlusion study

Thirty-six healthy volunteers (12 per parameter) were enrolled in a laboratory study at the Innovation Center Computer Assisted Surgery, Leipzig, Germany to evaluate the MSI sequences. The study was approved by the local ethics committee (476/21-ek) and is registered at clinicaltrials.gov (NCT05348512). Each participant gave written informed consent. Further information about the study cohort is available in Sec. 3.2 and Table S1 in the Supplementary Material.

**Fig. 3:**
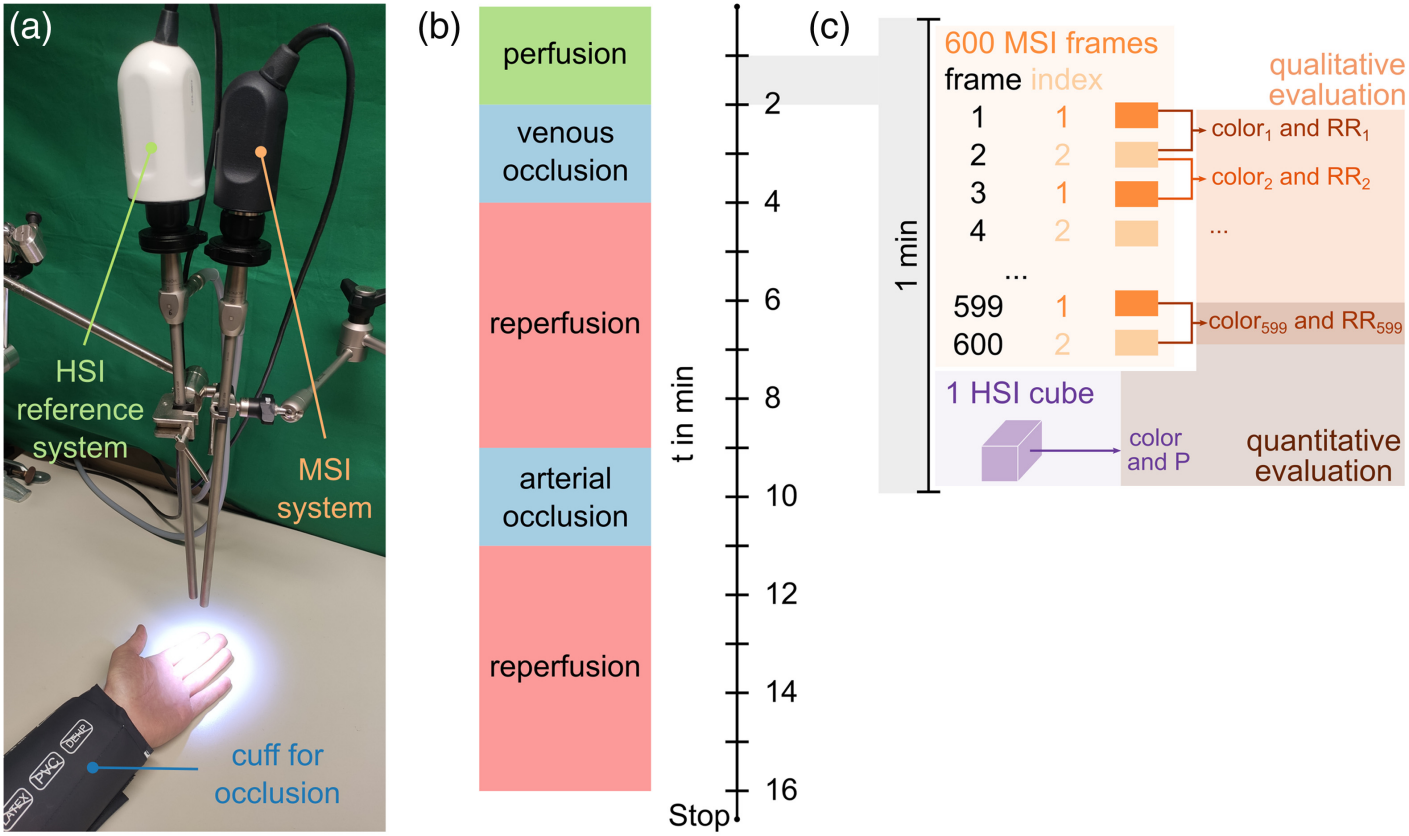
(a) Setup during the laboratory study with healthy volunteers in which HSI and MSI data were acquired according to the occlusion protocol (b). (c) Each minute, 600 MSI frames were recorded followed by one hypercube. Two consecutive MSI frames were used to calculate the RGB image (color) and an RR image. Reference color and parameter (P) images were calculated from the hypercube. All MSI color and RR images served for qualitative evaluations, i.e., parameter video calculations. The last MSI color and RR image, as well as the HSI color and P image, were used for quantitative evaluations, i.e., calibration and testing.

The overall setup of the study is shown in Fig. 3(a). The volunteers placed their left arm on a table while sitting on a chair. The participants were asked not to move during the measurement without using additional tools to stabilize their hands. A standard blood pressure cuff (Green Cuff superb, ERKA, Bad Tölz, Germany) was applied to the left wrist. The MSI system and the reference HSI system were fixed in holding arms (KARL STORZ SE & Co. KG, Tuttlingen, Germany) above the left hand. Both systems covered the same section of the left index and middle fingers.

The 16-min measurement was performed during 2 min of normal perfusion, 2 min of venous occlusion, 5 min of reperfusion, 2 min of arterial occlusion, and another 5 min of reperfusion [Fig. 3(b)]. The normal blood pressure was determined for each participant to individually adjust the applied occlusion pressures 5 min before the measurement started.

The acquired data per minute consisted of 600 MSI frames and a following HSI hypercube [Fig. 3(c)]. MSI color and RR images, as well as HSI color and parameter (P) images, were calculated from raw data. All MSI color and RR images per minute were used for qualitative analysis. The last MSI color and RR images per minute, as well as the HSI color and P images, served for quantitative evaluation (Sec. 2.4.3).

MSI data were recorded at distances of 35, 45, and 55 mm to reduce the distance dependency of parameter calculations for future handheld measurements. The working distance for the HSI system remained constant at 50 mm.

#### 2.4.3 Data processing and parameter evaluation

**Fig. 4:**
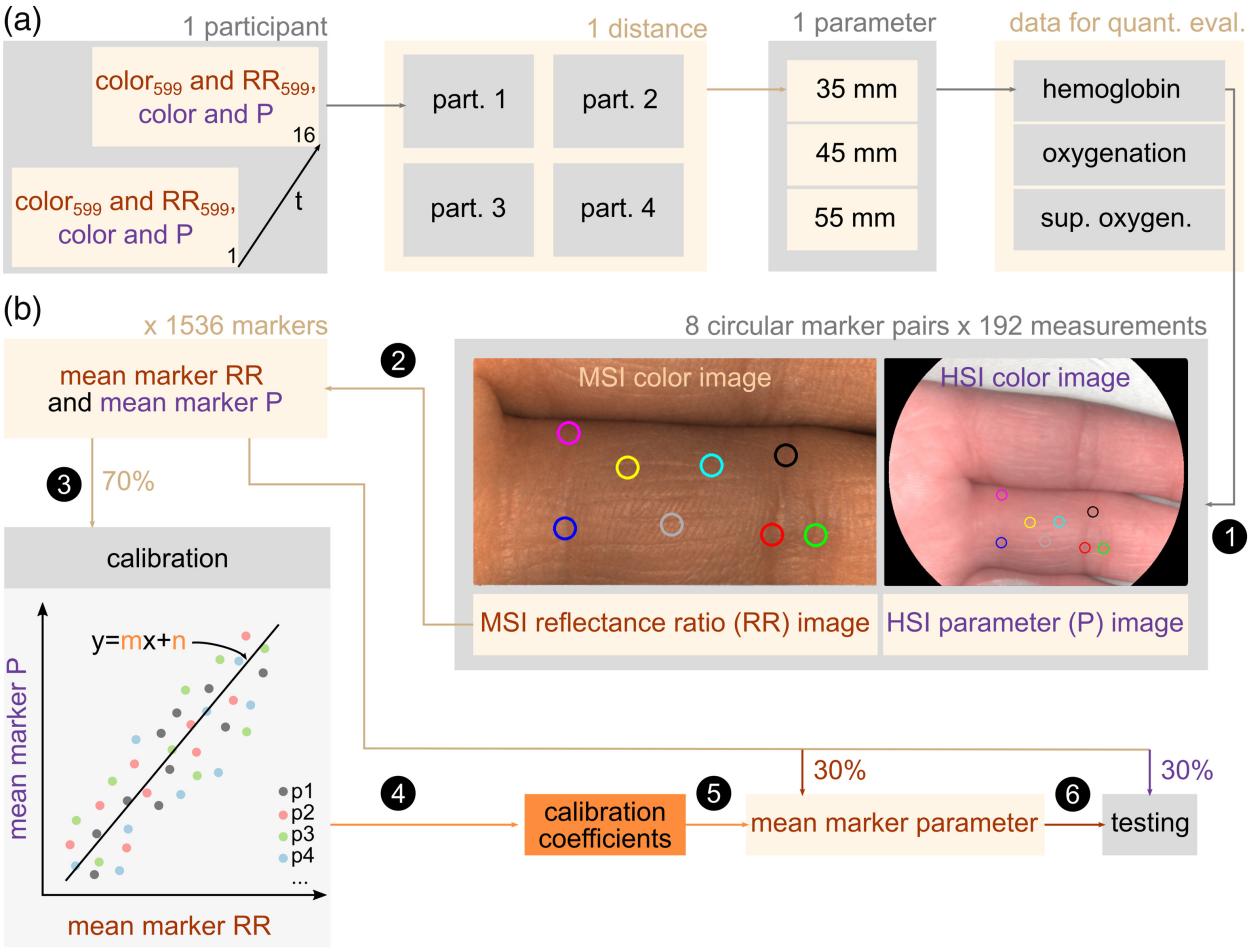
(a) The dataset for quantitative evaluations included 16 MSI color and RR images as well as 16 HSI color and P images per participant (also see Fig. 3). For each measurement distance, four participants were enrolled. Three measurement distances were selected for each parameter, resulting in 12 participants and 192 measurements per parameter. (b) Data processing. Step 1: each measurement per parameter (n=192) included MSI color and RR images as well as HSI color and P images. Up to eight circular markers were set in each image. Step 2: mean marker RR and mean marker P values were respectively calculated from MSI RR images and HSI P images. Step 3: 70% of 1536 mean marker RR and P value pairs (8 markers × 192 measurements) were used to train a linear regression model per parameter. Step 4: the regression coefficient and y-intercept served for the calibration of RR to P values. Step 5: mean marker parameter values were calculated from the remaining 30% of mean marker RR using the calibration coefficients. Step 6: calculated mean marker MSI parameters were quantitatively compared to corresponding mean marker HSI parameters.

After completing data acquisition, quantitative evaluations of 192 measurements were performed first. The total number of measurements is the product of the number of measurements per participant (m=16), the number of participants per measurement distance (p=4), and the number of measurement distances (d=3) [Fig. 4(a)].

Up to eight anatomically corresponding circular markers were manually set in each HSI-MSI data pair [Fig. 4(b), step 1]. The size of the markers was manually adjusted by imaging a circular reference object with both camera systems and measuring its pixel diameter. Reference values for tissue hemoglobin content (THI) and oxygenation (NIR PI and StO2) were calculated as mean marker values from the HSI parameter (P) image. Mean marker RRs were calculated from MSI RR images [Fig. 4(b), step 2]. The latter were calibrated to the HSI reference values by splitting up mean marker value pairs (HSI mean marker P and MSI mean marker RR) into calibration (70%) and test (30%) data. The Kolmogorov–Smirnov distance was minimized using the function “ks_2samp” from the statistics module of the Python package “SciPy” to guarantee a similar distribution of reference values in the calibration and test datasets. An overview of the reference mean marker values in both calibration and test datasets for each tissue parameter is given in Table S2 in the Supplementary Material.

The calibration dataset was used to train an ordinary least squares linear regression model per parameter with the Python function “sklearn.linear_model.LinearRegression().fit(RR, P)” [Fig. 4(b), step 3]. The models were evaluated by fivefold cross-validation. The regression coefficients and y-intercepts [Fig. 4(b), step 4] were used to calculate mean marker hemoglobin, oxygenation, and superficial oxygenation from the MSI RRs in the test dataset [Fig. 4(b), step 5]. They were quantitatively compared to reference THI, NIR PI, or StO2 in the test dataset [Fig. 4(b), step 6], respectively, by calculating the root mean squared error (RMSE), the coefficient of determination ($R^{2}$), and the residual prediction deviation (RPD)^16^ as the standard deviation of the reference data divided by the RMSE. RMSE, $R^{2}$, and RPD were determined for worst-case linear regression models with regression coefficients of 0 and y-intercepts equal to the mean reference THI, NIR PI, and StO2 in the calibration dataset. All quality metrics were related to the measurement distance and sex of participants.

Furthermore, the difference between each calculated mean marker MSI parameter and the corresponding mean marker reference HSI parameter was determined for each individual for intra- and interindividual analyses and compared to the age of the participants as well as to the mean marker SNR. The Mann–Whitney-U test was used to investigate if marker differences depend on the measurement distance or sex of participants with a significance level of p=0.05. The Python function “scipy.stats.mannwhitneyu” was used for this purpose. To do so, the eight dependent marker differences that belong to the same out of 16 measurements per participant were averaged previously by calculating the mean absolute error (MAE). This resulted in 16 independent MAE values per participant, which were included in the statistical test.

Finally, calibration coefficients were used to calculate MSI parameter images from all RR images [see Fig. 3(c)] according to Eq. (5) to be visualized as videos and qualitatively compared to expected parameter courses

$$P_{\mathrm{MSI}}=m\cdot {\mathrm{RR}}_{\mathrm{MSI}}+n,$$ (5)

with $P_{\mathrm{MSI}}$: MSI parameter value and m,n: calibration coefficients.

## 3 Results

This section covers the results of the technical evaluations of the overall system. A brief overview of the cohort included in the occlusion study and the acquired HSI reference data is given before the results of the tissue parameter calculation and visualization.

### 3.1 Technical Evaluation

Signals of LEDs were only measured in frames where LEDs had been turned on (image index 2) during LED switch-on studies. Neither transient response nor afterglow was observed [Fig. S2(a) in the Supplementary Material]. Pixel values [0, 1023] averaged over the three color channels, all LEDs, all measurements, and spatially were 307.5 for image index 2 and 1.3 for image indexes 1 and 3. The latter is related to background noise and is negligible. In continuous mode, i.e., an LED was turned on over all three images of a sequence, the intensities of all LEDs remained constant over one hour. Deviations to the initially measured intensity at t=0 were lower than 2%, proving the long-term stability of LEDs [Fig. S2(b) in the Supplementary Material]. A relation between calculated MSI tissue parameter deviations from reference HSI parameters and lighting fluctuations was therefore not assumed.

**Fig. 5:**
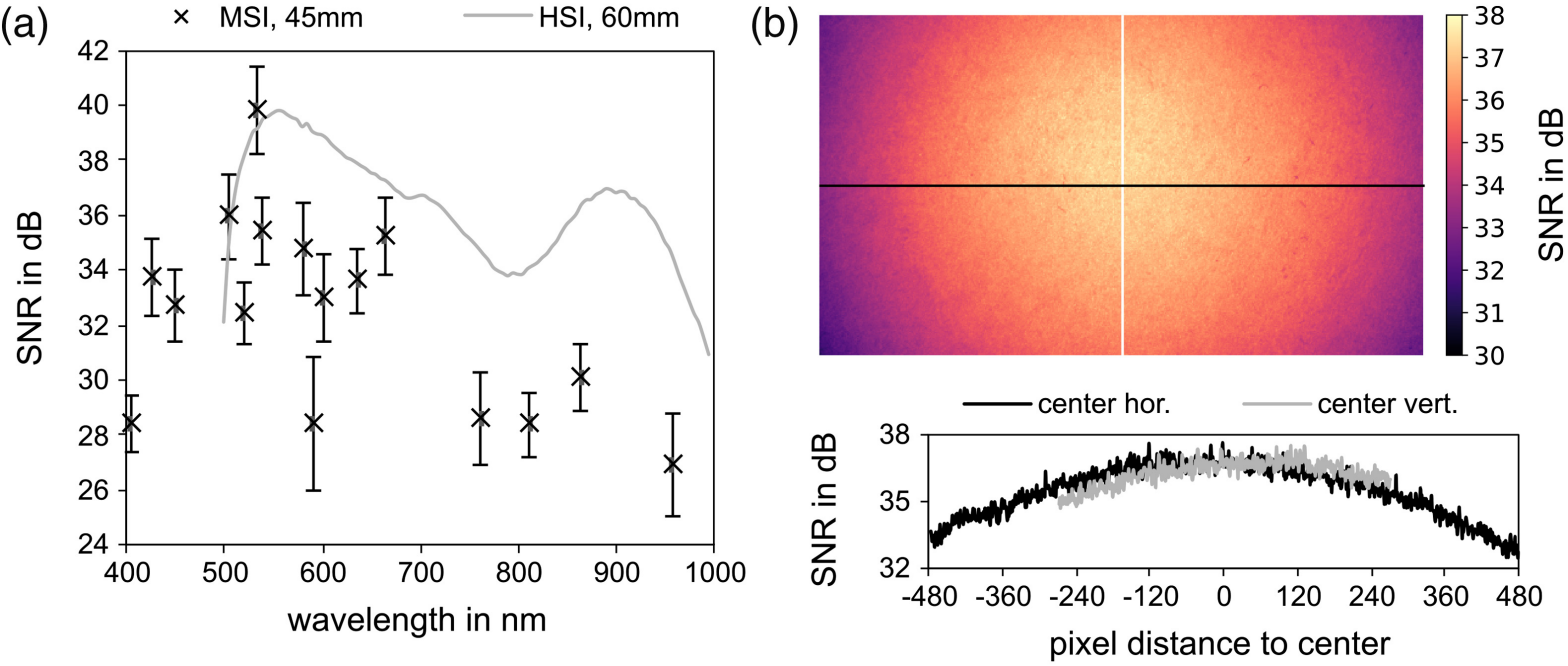
(a) Comparison of SNRs between the presented MSI system and the reference HSI system measured at optimal working distances for both systems. MSI values are provided as spatial mean and standard deviation. (b) Spatially resolved SNRs for one effective wavelength (537 nm) for the whole image area (top) as well as for the mean horizontal and vertical line (bottom).

SNRs varied between 26 and 40 dB. LEDs emitting in the NIR range performed worse (≤30  dB). SNRs were lower than SNRs of the reference HSI system at 519 nm, as well as wavelengths higher than 532 nm and similar at 503 and 532 nm [Fig. 5(a)]. Low SNRs in the red and NIR spectral ranges limit the resolution of small differences in tissue oxygenation and the application of the system at measurement distances larger than 55 mm.

The MSI system achieved SNRs between 28 and 34 dB in the blue spectral range, which is of interest for a natural color image or future developments concerning contrast-enhanced imaging of blood vessels. The reference system does not provide data in the blue spectral range.

Pixel-wise SNRs are exemplarily visualized for the effective wavelength of 537 nm as a heatmap for the whole image area as well as line plots for the mean horizontal and mean vertical pixel line in Fig. 5(b). SNRs decreased from the image center to the edge by approximately 4 and 2 dB along the horizontal and vertical axis, respectively. The reduction of the SNRs along one axis is asymmetrical to the center of the image, which indicates a slightly off-center illumination.

### 3.2 Occlusion Study

The total occlusion study cohort consisted of 22 male and 14 female participants. The median age was 32, ranging from 18 to 52 years. An extended overview of the distribution of participants over the three tissue parameters and different working distances of the MSI system is available in Table S1 in the Supplementary Material. The data of one participant (hemoglobin content at 35 mm) had to be discarded because of invalid MSI pixel values (sensor override).

Reference tissue parameters [0, 1] acquired with the HSI system respectively varied between 0.031 and 0.907, 0.400 and 0.797 as well as 0.274 and 0.881 for THI, NIR PI, and StO2 (Table S2 in the Supplementary Material). The StO2 was mainly high (mode≈0.8). Lower values were acquired during the venous or arterial occlusion only and varied little across subjects. The variation of NIR PI values mainly came from the inclusion of different subjects rather than from the occlusion. Both occlusion and diverse subjects contributed to the broad range of values for the THI. The means and standard deviations of all reference values across the different states of perfusion are depicted in Fig. S3 in the Supplementary Material.

### 3.3 Tissue Hemoglobin and Oxygenation Imaging

MSI RRs were calibrated to HSI reference values from the calibration dataset through linear regression models (Sec. 2.4.3). According to the results after fivefold cross-validation, the model for tissue oxygenation performed best (Table 2). The model for superficial tissue oxygenation hardly differed from the worst-case scenario (RMSE 0.09 versus 0.11). The model verification using the test dataset led to similar results. Across all participants, the tissue oxygenation could be calculated with the lowest RMSE. Neither distance nor sex-related dependencies of the RMSE were observed. There were higher variations in $R^{2}$ and RPD values due to different value distributions in subclasses (Table 2). MAEs did not differ significantly between males and females (p=0.444), measurements at 35 and 45 mm (p=0.249), and measurements at 45 and 55 mm (p=2.05). However, there was a significant difference between measurements at 35 and 55 mm (p=0.031) [also see Fig. S4(a) in the Supplementary Material], which is related to the deviation from the optimum lighting settings (image sensor gain and LED switch-on time) defined for a working distance of 45 mm.

**Table 2:** Resulting metrics after fivefold cross-validation (calibration dataset) and verification (test dataset) of linear regression models for each parameter compared to the worst-case scenarios. The coefficient of determination ($R^{2}$), the RMSE, and the RPD were calculated for the whole cohort as well as subclasses (distance- or sex-related).

Parameter	i.tHb			i.StO2			${\mathrm{i.StO}2}_{\sup}$			
Cohort	$R^{2}$↑	RMSE↓	RPD↑	$R^{2}$↑	RMSE↓	RPD↑	$R^{2}$↑	RMSE↓	RPD↑	
Calibration (fivefold cross-validation)										
All	0.65	0.08	1.69	0.75	0.04	2.01	0.43	0.09	1.33	
Testing										
All	0.68	0.07	1.77	0.77	0.04	2.09	0.42	0.09	1.32	
35 mm	0.49	0.08	1.40	0.66	0.04	1.71	0.51	0.07	1.42	
45 mm	0.55	0.08	1.50	0.78	0.04	2.12	0.18	0.10	1.10	
55 mm	0.78	0.06	2.12	0.74	0.03	1.98	0.25	0.11	1.15	
male	0.71	0.08	1.85	0.75	0.04	2.01	0.46	0.09	1.36	
female	0.61	0.07	1.60	0.57	0.04	1.52	0.38	0.10	1.28	
Worst-case scenario										
—	0.00	0.12	1.00	0.00	0.09	1.00	0.00	0.11	1.00	

The model for tissue hemoglobin content did not show any dependencies of the RMSE on distance or sex (Table 2). The worse performance compared to the model for i.StO2 might originate from the broader range of reference values in the hemoglobin calibration and test datasets, which has to be investigated in future trials. In addition, information from the blue and red spectral range, which is simultaneously acquired to calculate a color image, could distort the green information used to calculate the hemoglobin parameter more than is the case with the oxygenation parameter (Fig. 2). Similar to the results of the oxygenation parameter, MAE did not differ with the sex of participants (p=0.886). A significant dependency on the working distance [Fig. S4(a) in the Supplementary Material] was determined on the other hand for 35 versus 55 mm (p=0.0002) and 45 versus 55 mm (p=0.0004). There was no significant difference between 35 and 45 mm (p=0.783). However, it cannot be assumed that the parameter error increases with increasing working distance, as the RMSE was lowest at 55 mm (Table 2).

The worse performance of the model for the superficial oxygenation was traced back to the accumulation of high HSI reference values and a poor representation of lower values in the calibration and test datasets. Furthermore, the low spectral resolution of the MSI system in the green spectral range (FWHM of LEDs range from 32 to 75 nm), where the ratio of absorption coefficients of HbO2 and HHb changes within a few nanometers, must be considered. An additional distance dependency of the RMSE (Table 2) leads to the conclusion that the chosen illumination and the trained model are not suitable for the calculation of superficial oxygenation. The distance dependency was substantiated by significances [Fig. S4(a) in the Supplementary Material] between 35 and 45 mm (p<0.0001) as well as 35 and 55 mm (p=0.0016). There was no significant difference between 45 and 55 mm (p=0.253) or males and females (p=0.635).

The model for i.StO2 also performed best when considering intra- and interindividual variations [Fig. S4(b) and Table S3 for numerical values in the Supplementary Material]. Fig. S4(b) in the Supplementary Material furthermore shows that calculated MSI parameters for one participant can be both higher and lower than the reference HSI parameters. Besides sex or measurement distance, no relation between parameter errors and the age of participants or the SNRs of used spectral ranges was observed [Figs. S4(c) and S4(d) in the Supplementary Material].

Examples of resulting false-color parameter images without any additional image postprocessing can be seen in time-lapse Videos 1–3 in the Appendix. The higher spatial resolution is visible at a limited field of view in the MSI videos in contrast to the HSI reference images. Different perfusion states but also pixel-wise deviations from the HSI system are recognizable. Glare artifacts mainly appeared at a working distance of 35 mm and dark image areas at 55 mm because the light intensity was adjusted to 45 mm.

## 4 Discussion and Conclusion

This work presents and technically evaluates a laparoscopic MSI system based on 14 switchable LEDs in the VIS and NIR spectral range. A data acquisition and processing pipeline were developed to calculate and visualize tissue parameters in high spatial resolution and as video. An occlusion study with healthy volunteers was conducted for the calibration to a reference HSI system and to evaluate the whole pipeline. Deviations from the reference system were acceptable. Improvements in hardware and software as well as extended (pre-) clinical trials are necessary, which are mentioned in the following sections.

### 4.1 Technical Improvements

Light source requirements such as available spectrum, long-term stability, switching behavior, or synchronization with the image sensor were fulfilled. Although comparable filter wheel^4^ and snapshot^17^ systems respectively reach higher spectral resolutions (FWHM: 10-25 nm versus 12 to 75 nm) and temporal resolutions (frame rate: 25 fps versus 20 fps), the presented MSI system benefits from a broader spectral range beyond the visible range and carries the potential for further applications like tissue discrimination.

The final frame rate, the field of view, and the image size are currently limited by the internal data transfer from the image sensor via an NVIDIA Jetson server to an integrated Windows system. By directly performing the raw data processing on the Jetson server, the full potential of the image sensor (4K images with 60 fps) will be exploited. Using two consecutive raw images to calculate one tissue parameter image, the effective frame rate will then rise to 30 fps.

Light coupling into the fiber must be improved because SNR measurements indicated a slightly off-center illumination and low light intensities in the NIR range. Condensers, ray trace simulations to design optimized multi-branched light guides,^18^ or alternative LED arrangements like a ring layout are methods of choice. For the latter, it was recommended to use multiple LEDs of each wavelength.^19^ Systems have been presented with identical LEDs opposite to each other^20^ or arranged 90 deg apart from each other.^20^^,^^21^ The results will be lower light losses and better mixing of individual wavelengths, the ability to visualize smaller gradients of tissue hemoglobin content or oxygenation, and the use of the system at larger measuring distances. However, multiple identical LEDs will be associated with a reduction of the available spectral range, higher costs, or bulky setups. Alternatively, temporal averaging as performed in Ref. 10 can increase the SNR without further hardware adaptations but leads to reduced frame rates.

Current SNR values did not affect the parameter errors within the chosen working distance of 35 to 55 mm and are higher than 25 dB. A cut-off value of 20 dB was determined by Liu et al.^21^ in investigating the contribution of spectral channels to the accuracy of tissue classification algorithms depending on their SNR. Similarly, Mühle et al.^22^ discarded spectral information with SNR values being lower than 25 dB. An SNR threshold for perfusion imaging has to be defined in future trials comparing the presented MSI system and the reference HSI system at larger measurement distances. However, for currently chosen distances, the SNR values were acceptable as the resulting parameter errors were acceptable.

After technical improvements, further optical characteristics, e.g., the influence of the light guide cable on the illumination must be determined. So far, the position of the light guide cable remained constant during all measurements.

### 4.2 Occlusion Study

The performed occlusion study mainly included young adults. Acquired reference data to calibrate the MSI system could not cover a broad range of tissue oxygenation and hemoglobin content values in all cases. The data acquisition was extracorporeal only. The significance of presented results for future surgical applications like colorectal, gastric, or esophageal resections is therefore limited. Measurements on perfused, insufficient perfused organs, and in elderly and diseased persons will be part of subsequent trials. Possible dependencies on further distances or angles of illumination will also be investigated. Motion compensation algorithms are going to be included in the data processing pipeline to enable perfusion imaging in handheld laparoscopic settings.

Nevertheless, calibration and comparison of the developed MSI system to the reference HSI system within the occlusion study suggest applicability in the intended laparoscopic use case, as the reference system is already used in visceral surgery without any adaption of data collection, processing, and oxygenation calculation. In contrast, as shown in Ref. 11, the tissue hemoglobin parameter cannot be applied directly to measurements on organs. A scaling factor of 0.5 between tissue and organ hemoglobin parameters was defined. The translatability of this relationship to the MSI system needs to be demonstrated in further studies.

### 4.3 Tissue Hemoglobin and Oxygenation Monitoring

The presented imaging sequences and the preceding wavelength selection for tissue hemoglobin content and oxygenation monitoring were based on the absorption characteristics of oxygenated and deoxygenated hemoglobin. Although the spectral ranges used are similar to those of Ref. 9 (470 and 530 nm versus 450, 532, and 537 nm for hemoglobin), in Ref. 23 (660 and 880 nm versus 663, 811, and 863 nm for oxygenation), and the TIVITA Mini, the use of additional or alternative wavelengths might result in improved measurements. Tissue modeling and simulated MSI^24^^–^^26^ as well as partial least squares regression^14^^,^^27^^–^^29^ are promising wavelength selection methods and will be part of future investigations.

A quantitative comparison between presented hemoglobin content and oxygenation maps and competing laparoscopic MSI systems is currently limited. Intraoperatively tissue perfusion is measured by clinical assessment lacking an objective gold standard. MSI has therefore been evaluated qualitatively.^4^^,^^17^ *In-vivo* animal studies in which bowel tissue oxygenation is selectively reduced or in which tissue perfusion is imaged by fluorescence angiography should be performed in the future to investigate the added value and accuracy of MSI perfusion monitoring.

## 5 Appendix: Supplementary Information

1 Video 1. Calculated i.tHb parameter images at 45 mm compared to the reference THI during different perfusion states (MP4, 11 MB [URL: https://doi.org/10.1117/1.JBO.28.12.126002.s1]).
2 Video 2. Calculated i.StO2 parameter images at 35 mm compared to the reference NIR PI during different perfusion states (MP4, 11.7 MB [URL: https://doi.org/10.1117/1.JBO.28.12.126002.s2]).
3 Video 3. Calculated i.StO2sup parameter images at 55 mm compared to the reference StO2 during different perfusion states (MP4, 11.8 MB [URL: https://doi.org/10.1117/1.JBO.28.12.126002.s3]).

## Data Availability

Code associated with this paper is subject to confidentiality between the project partners and cannot be made available. A dataset was generated during the occlusion study with healthy volunteers. It includes hyperspectral and multispectral images, age, sex, skin type, and normal blood pressure. The dataset is not available for externals. Diaspective Vision GmbH and KARL STORZ SE & Co. KG provided the hardware to perform the presented study. The further transfer of materials is not possible.
