# Supplementary material for: Switchable LED-based laparoscopic multispectral system for rapid high-resolution perfusion imaging
Source: J Biomed Opt. 2023 Dec 13;28(12):126002. doi: 10.1117/1.JBO.28.12.126002 (PMC10718192; doi:10.1117/1.JBO.28.12.126002)
Supplement: Supplementary file 1 [file JBO_028_126002_SD001.pdf]

Switchable LED-based Laparoscopic Multispectral System for Rapid High-Resolution Perfusion Imaging

Annekatrin Pfahl,[a,*] Süleyman T. Polat,[a] Hannes Köhler,[a] Ines Gockel,[b] Andreas Melzer,[a,c] Claire Chalopin[a,d]
[a]Leipzig University, Faculty of Medicine, Innovation Center Computer Assisted Surgery (ICCAS), Semmelweisstrasse 14, Leipzig, 04103, Germany
[b]University Hospital of Leipzig, Department of Visceral, Transplant, Thoracic, and Vascular Surgery, Liebigstrasse 20, Leipzig, 04103, Germany
[c]University of Dundee, School of Medicine, Institute for Medical Science and Technology (IMSaT), 1 Wurzburg Loan, Dundee, DD2 1FD, United Kingdom
[d]University of Applied Sciences and Arts, Faculty of Engineering and Health, Von-Ossietzky-Strasse 99, Göttingen, 37085, Germany

Appendix A: Supplemental Material

In this appendix, additional material analyzing the multispectral laparoscope and the performed occlusion study is available.

The spatial resolution of the whole system was investigated with the STEMMER IMAGING test chart for machine vision. Therefore, the Michelson contrast was determined for six line patterns with object resolutions from 1 lp/mm to 10 lp/mm at a working distance of 45 mm using an objective lens with a focal length of 25 mm and with three illumination patterns. The illumination patterns were designed to measure and visualize the superficial oxygenation (i.StO2$_{sup}$), oxygenation in deeper layers (i.StO2), and hemoglobin content (i.tHb). Defining a lower contrast limit of 20 %, the spatial resolution of the MSI system is 3.2 lp/mm (156 µm) in all three cases (Fig. S1).

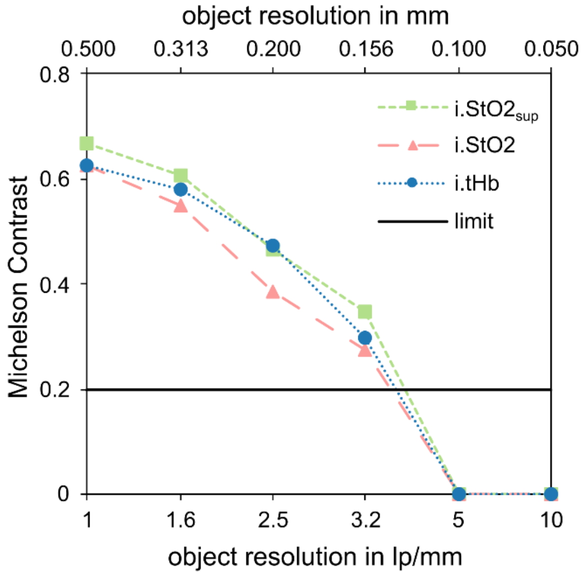

Fig. S1 Test chart object resolution in lp/mm and mm (x-axes) and resulting Michelson contrasts for three different illumination patterns.

The LED switching behavior and long-term stability were investigated for further technical evaluations of the laparoscopic MSI system. Raw data is visualized in Fig. S2. A description of the underlying measurements, results and conclusions can be found in the main body of the publication (Sec. 2.2, 3.1, and 4.1).

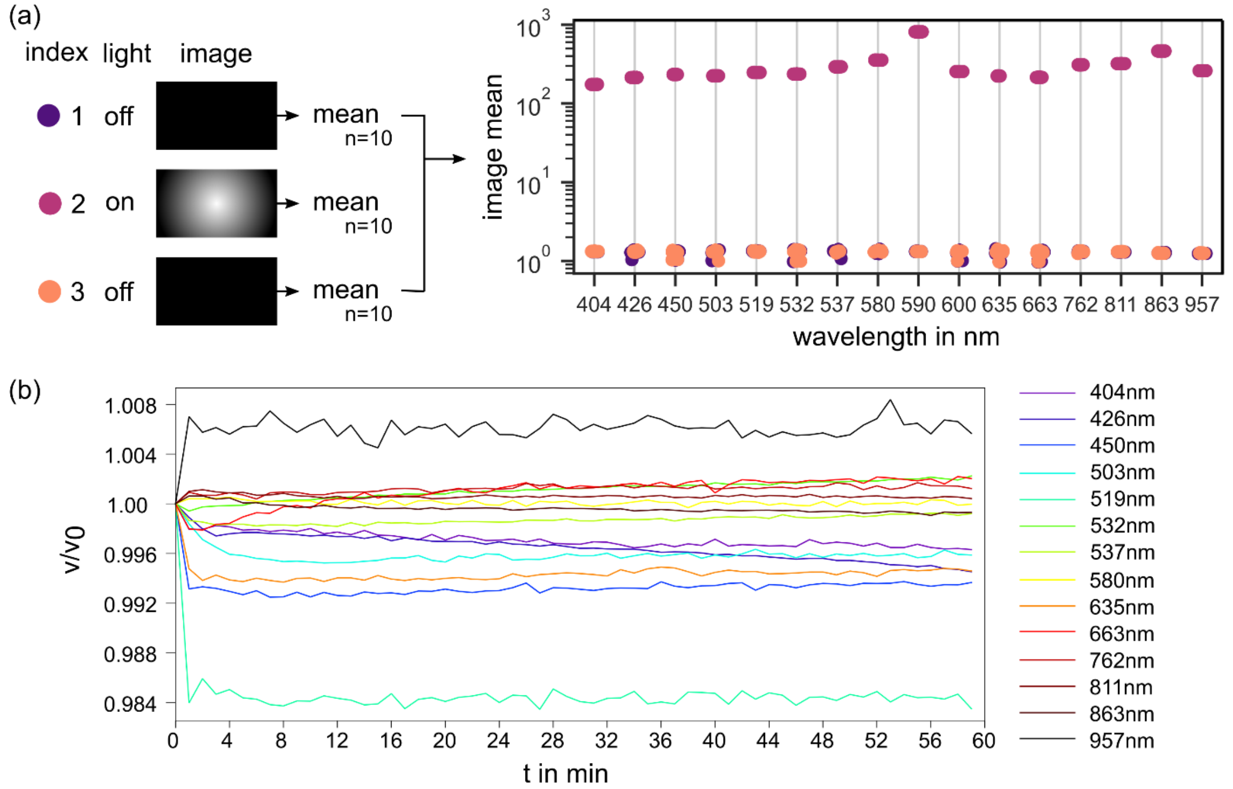

Fig. S2 (a) The switching behavior of LEDs was determined by defining an illumination sequence in which the considered LED was off within images 1 and 3, and on within image 2. For each image index, the pixel value was spatially averaged and calculated over 10 frames. (b) Visualization of the long-term stability of each LED. Each minute, the mean pixel value of the actual frame (v) was related to the first frame at t=0 (v_0).

Reference values were acquired with the TIVITA Mini (Diaspective Vision GmbH, Am Salzhaff-Pepelow, Germany) during the occlusion study. This laparoscopic hyperspectral imaging system provides tissue parameters like superficial oxygenation (StO2), oxygenation in deeper layers (NIR PI short for near-infrared perfusion index), and hemoglobin content (THI short for tissue hemoglobin index). A timeline of recorded data is presented in Fig. S3.

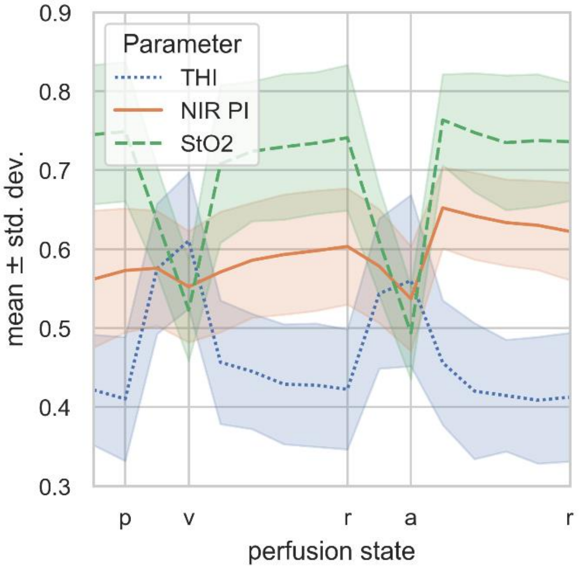

Fig. S3 Timeline of HSI reference values during the occlusion study averaged over all participants. The broadest range of values was achieved for the THI, followed by the StO2 and NIR PI. The x-axis indicates the endpoints of normal perfusion (p, after 2 min), venous occlusion (v, after additional 2 min), reperfusion (r, after additional 5 min), arterial occlusion (after additional 2 min), reperfusion (after additional 5 min).

An overview of the study cohort is given in Table S1. In total, 36 healthy volunteers (12 per parameter) were enrolled. For each parameter, they were split up into three groups of four individuals. In each group, a certain working distance of the multispectral imaging system was used, ranging from 35 to 55 mm.

Table S1 Occlusion study cohort for the calibration and evaluation of the presented system.

**parameter**	**all**	**i.tHb**				**i.StO2**				**i.StO2$_{sup}$**			
distance in mm	all	all	35	45	55	all	35	45	55	all	35	45	55
n	36	12	4	4	4	12	4	4	4	12	4	4	4
sex													
male	22	7	2	3	2	9	3	4	2	6	2	2	2
female	14	5	2	1	2	3	1	0	2	6	2	2	2
age													
median	32	38	35.5	37	41	33	32.5	34	33.5	28	28	30.5	26.5
min	18	29	32	29	30	23	25	31	23	18	22	24	18
max	52	52	43	42	52	38	36	38	38	33	33	32	29

In Table S2, the median, modal, minimum, and maximum of the acquired reference tissue parameters with a distinction between calibration and test datasets are listed. The THI comprised the largest value range, followed by the StO2 and NIR PI. For each parameter, Kolmogorov-Smirnov distances of approximately 0.03 indicate a similar distribution of reference values in both datasets. The largest number of calibration and test values was available for the StO2 (n=1535), followed by the NIR PI (n=1530) and THI (n=1399). Since reference values were extracted from eight circular regions of interest for each of the 16 records across 16 minutes per individual and a total amount of 12 participants per parameter, the maximum number of reference values per

parameter was 1536. Some regions of interest and thus reference values had to be discarded due to glare or motion artifacts.

Table S2 Reference tissue parameter values from the HSI system in the calibration and test datasets acquired during the occlusion study, and amount of data included in the whole cohort as well as distance- or sex-related.

**parameter**		**THI**		**NIR PI**		**StO2**	
	limit	calib	test	calib	test	calib	test
Kolmogorov-Smirnov distance	[0;1]	0.025		0.023		0.025	
n	1536	979	420	1071	459	1074	461
median	[0;1]	0.46	0.46	0.60	0.60	0.73	0.73
mode	[0;1]	0.44	0.41	0.49	0.64	0.77	0.70
min	0	0.06	0.03	0.40	0.40	0.29	0.27
max	1	0.91	0.89	0.80	0.79	0.88	0.88
n @35mm		263	121	356	150	360	151
n @45mm		357	146	349	163	361	151
n @55mm		359	153	366	146	353	159
n females		354	158	275	109	531	237
n males		625	262	796	350	543	224

Fig. S4 shows raw data that was used for statistical tests (a), intra- and interindividual comparisons (b), and to investigate the dependency of the parameter error on the age of participants or the signal-to-noise ratio (SNR).

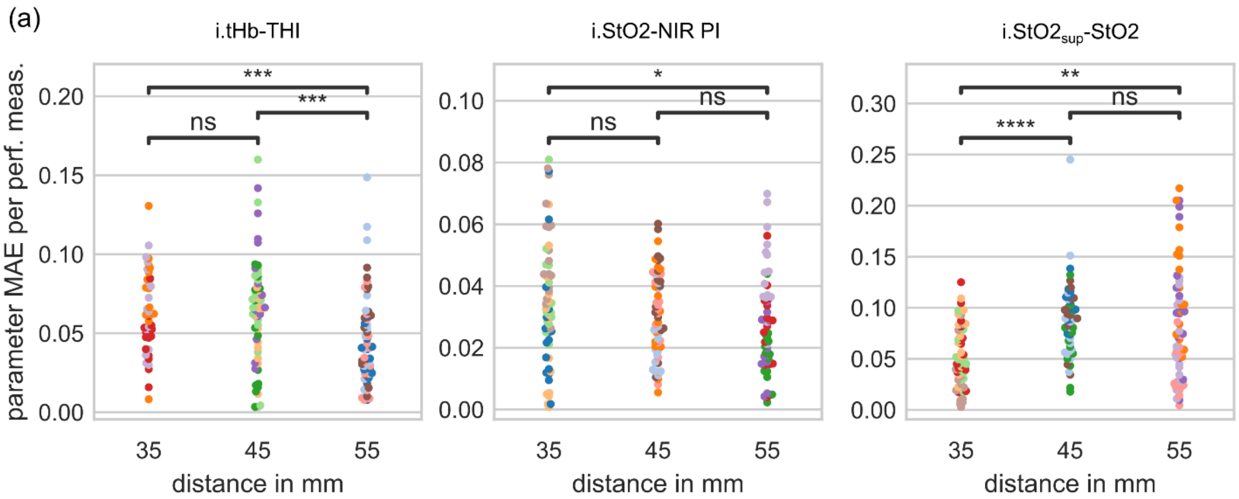

Fig. S4 (a) Mean absolute errors (MAE) for each perfusion measurement (16) between mean marker MSI and HSI parameters depending on measurement distances.

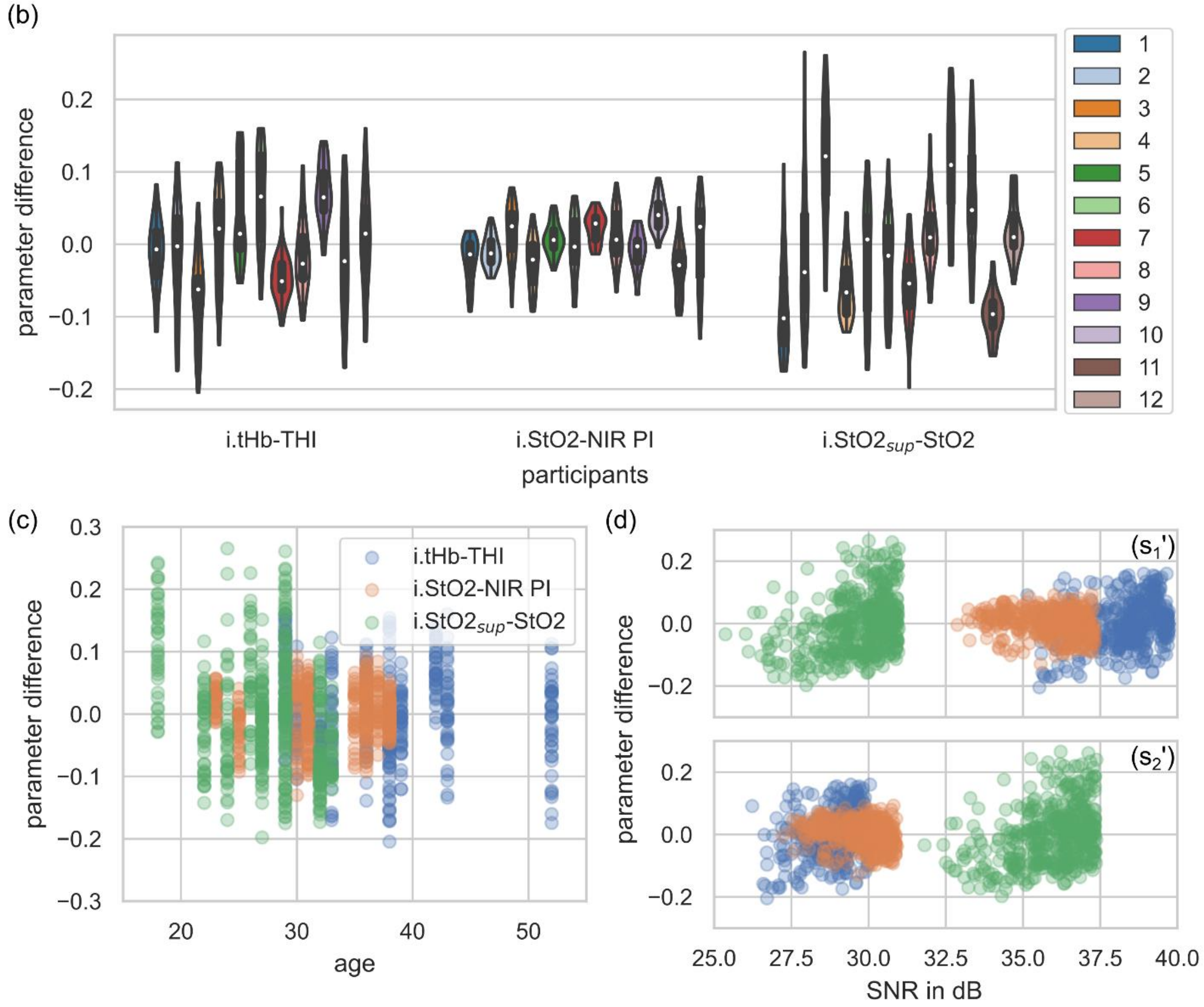

Fig. S4 (cont.) Marker difference for each participant (b), depending on the age of participants (c), and the mean marker SNR in spectral ranges $\boldsymbol{s}_1'$ and $\boldsymbol{s}_2'$ (d). The legend in (b) also applies to (a). The legend in (c) also applies to (d). In (a), significances of the Mann-Whitney-U test are symbolized as non-significant (ns), p<0.05 (*), p<0.01 (**), p<0.001 (***), and p<0.0001 (****). The violins in (b) represent the distribution of parameter differences for all circular markers (8 markers x 16 measurements per participant, see also Fig. 4b). White circles indicate median values. Black boxes are limited by the first and third quartiles.

Table S3 shows intra- and interindividual variations of parameter differences (calculated MSI parameter minus reference HSI parameter). It is related to Fig. S4b.

Table S3 Intra- and interindividual parameter differences. IQR – interquartile range.

**parameter difference**	**i.tHb - THI**		**i.StO2 - NIR PI**		**i.StO2$_{sup}$ - StO2**	
participant	median	IQR	median	IQR	median	IQR
1	*-0.01*	0.06	*-0.01*	0.04	-0.10	0.09
2	**0.00**	0.09	*-0.01*	*0.03*	-0.04	0.13
3	-0.06	0.07	0.03	0.05	0.12	0.11
4	0.02	0.10	-0.02	0.05	-0.07	0.06
5	*0.01*	0.12	*0.01*	**0.02**	**0.01**	0.13
6	0.07	0.11	**0.00**	0.06	*-0.02*	0.10
7	-0.05	**0.04**	0.03	*0.03*	-0.05	0.06
8	-0.03	0.06	*0.01*	0.05	**0.01**	0.05
9	0.07	*0.05*	**0.00**	*0.03*	0.11	0.11
10	-0.02	0.12	0.04	*0.03*	0.05	0.10
11	0.02	0.09	-0.03	0.05	-0.10	**0.03**
12	discarded		0.02	0.09	**0.01**	*0.04*

Videos S1-3 depict parameter videos for three individuals (one per tissue parameter). They were calculated after the data acquisition and recorded in time-lapse mode.

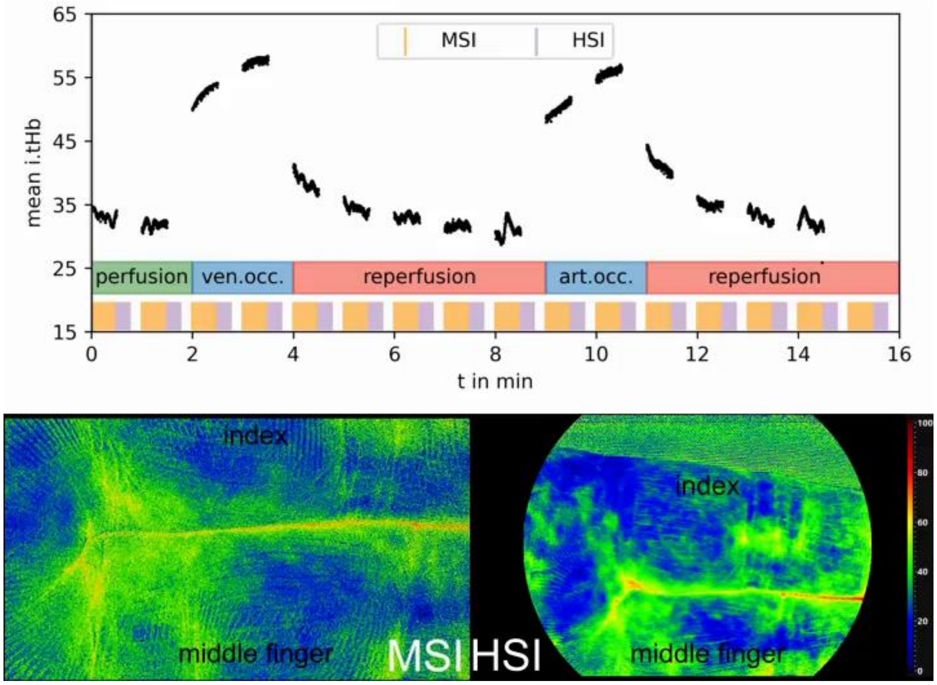

Video S1 Calculated i.tHb parameter images at 45 mm compared to the reference THI during different perfusion states (MP4, 11 MB).

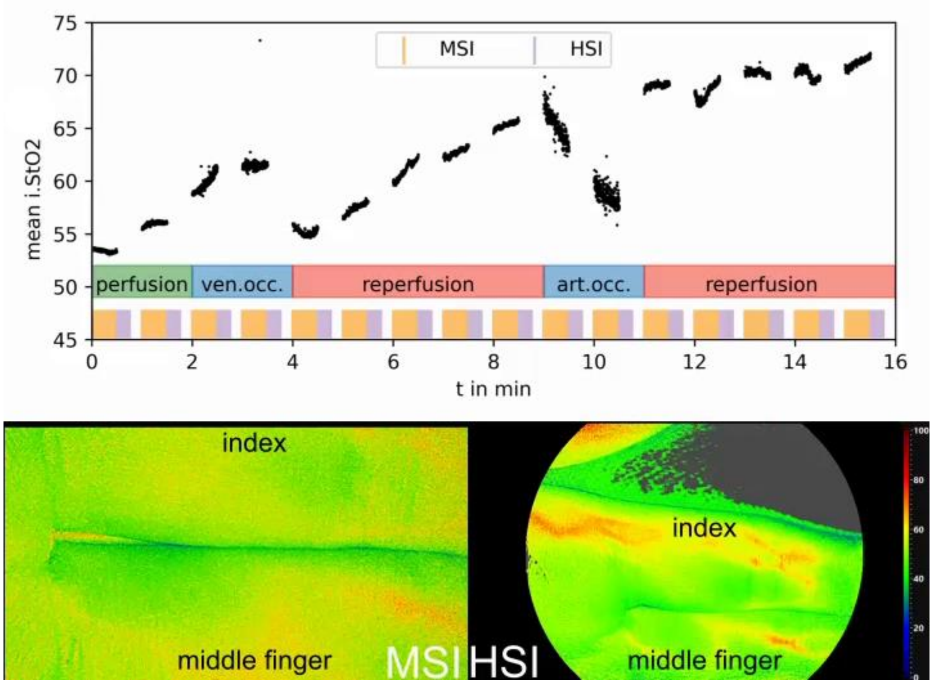

Video S2 Calculated i.StO2 parameter images at 35 mm compared to the reference NIR PI during different perfusion states (MP4, 11.7 MB).

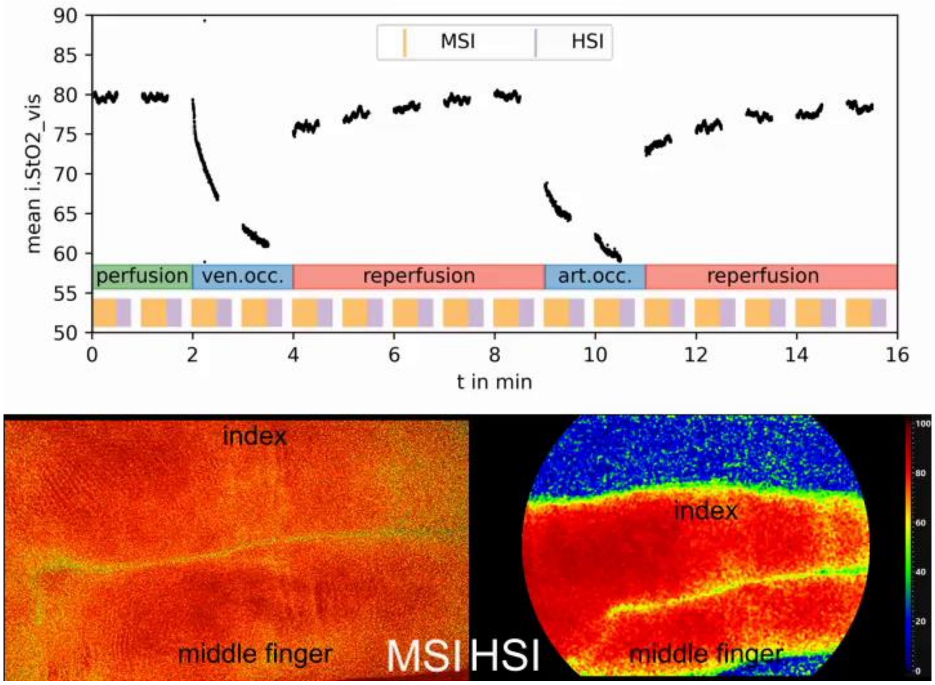

Video S3 Calculated i.$StO2_{sup}$ parameter images at 55 mm compared to the reference StO2 during different perfusion states (MP4, 11.7 MB).
